# Superior cognitive goal maintenance in carriers of genetic markers linked to reduced striatal D2 receptor density (C957T and DRD2/ANKK1-TaqIA)

**DOI:** 10.1371/journal.pone.0201837

**Published:** 2018-08-20

**Authors:** Jonas Persson, Cecilia Stenfors

**Affiliations:** 1 Aging Research Center, Karolinska Institutet and Stockholm University, Solna, Sweden; 2 Stress Research Institute, Stockholm University, Stockholm, Sweden; University of Queensland, AUSTRALIA

## Abstract

Maintaining goal representations is a critical component of cognitive control and is required for successful performance in many daily activities. This is particularly important when goal-relevant information needs to be maintained in working memory (WM), updated in response to changing task demands or internal goal states, and protected from interference by inhibiting counter-goal behaviors. Modulation of fronto-striatal dopamine is critical for updating and maintaining goals and representations. Here we test the hypothesis that a genetic predisposition (C957T T+ and DRD2/ANKK1-TaqIA A+) for reduced striatal D2 receptor availability would facilitate goal maintenance using the AX-continuous performance task (AX-CPT), on a sample of 196 adults (25–67 y). We demonstrate that carriers of two polymorphisms that have been linked to reduced striatal D2 receptor density show increased performance on context-dependent (BX) trials, and that the effect of these polymorphisms was only significant for long ISI trials where the demand for goal maintenance is high. The current results add further knowledge to the role of D2 receptor functioning in cognitive stability and flexibility, and could have implications for understanding cognitive deficits in patients characterized by altered dopamine functioning.

## Introduction

Goal directed behavior requires individuals to accurately balance between maintaining task-relevant information and update information when the context changes. To achieve this, it is necessary to form and maintain a top-down attentional set that align with environmental demands or an individuals’ internal state or goal. Goal maintenance refers to the processes associated with representation and active maintenance of goals or contexts based on internal or external cues necessary for appropriate behavior [1,2]. Goal maintenance and flexible updating has typically been investigated using working memory (WM) or continuous performance tasks (CPT; [3–5]).

Extant evidence indicate a role for dopamine (DA) in WM [6–8]. For example, several neuroimaging studies have pointed to the importance of prefrontal dopamine in WM by demonstrating dopamine drug related modulation of prefrontal BOLD signal during WM tasks [9–13]. While the exact mechanisms by which DA affects WM is still largely unknown, it has been suggested that DA enhances neural firing signal-to-noise ratio in the PFC [14] resulting in more stable goal-relevant representations (i.e. higher goal maintenance; [15–17]). While DA modulation in the PFC might be critical for stabilization and goal maintenance, in many situations there is a need for shifting attention and flexibly updating current goals and representations. Accumulating evidence suggest that DA is implicated in flexible updating and that this function is modulated by processing in the striatum. It has been suggested that tonic inhibitory input from the striatum to thalamus is involved in selective gating of motor programs [18], and there is evidence of a role for the striatum in updating of cognitive representations [19–22]. Individual differences in frontal vs. striatal dopamine regulation might therefore regulate to what extent an individual adopts maintenance or selective updating as a strategy for goal-relevant behavior.

In humans, the C957T (rs6277) single nucleotide polymorphism (SNP) of the D2 receptor gene (DRD2) affects messenger RNA (mRNA) stability. In particular, the T allele of the C957T SNP has been linked to reduced mRNA stability, affecting D2 receptor density in the striatum [23]. This allele has also been associated with reduced extrastriatal D2 receptor availability [24] and lower striatal DA levels [25]. In addition, the A1 allele of the Taq1A polymorphism (rs1800497) of the DRD2/ANKK1 gene has recently been the focus of much research. The A1 allele of the Taq1A polymorphism is located in the ANKK1 gene approximately 10kb downstream of the DRD2 gene. Reduced DA D2 receptor density [26,27] and binding [28] in the caudate nucleus and putamen has been demonstrated in carriers of the A1 allele of the DRD2/ANKK1-TaqIA polymorphism, and a recent meta-analysis has also linked this particular allele to reduced striatal D2 receptor availability [29]. Despite much evidence suggesting that these SNPs are strongly linked [23,30], few studies have systematically examined their effect in combination.

It has been hypothesized that striatal DA levels influence cognitive flexibility and stability, and that individual differences in these abilities are related to the DRD2/ANKK1-TaqIA allele. This is supported by demonstrations that A-carriers of the DRD2/ANKK1-TaqIA allele, who have less available striatal dopamine and presumably would show higher cognitive stability, perform worse on tests of working memory and task switching abilities [31–33]. Persson and colleagues [34] recently showed that, compared to non-carriers, older A carriers have worse memory updating performance, and exhibited less blood oxygen level-dependent (BOLD) activation in caudate nucleus while performing an updating task. This indicates that striatal activation mediates the link between the DRD2/ANKK1-TaqIA polymorphism and cognitive updating. The DRD2/ANKK1-TaqIA polymorphism was also recently linked to updating of mental representations in epistatic interaction with the COMT Val108/158Met genotype [35].

While there is much evidence for a link between the C957T polymorphism and cognitive phenotypes, the results are inconsistent with regards to the direction of effects. A few studies, however, indicate that carriers of the T allele show increased cognitive stability. For example, it has been shown that older DRD2 C957T T+ individuals are more able to inhibit a behavioral response to a stop signal [36]. In addition, studies on cognitive flexibility have demonstrated that carrying the DRD2 C957T T allele is a strong predictor of learning from negative reward prediction errors by avoiding responses linked to negative outcomes (i.e. "NoGo" learning; [37,38]). This indicate that the T allele of the DRD2 C957T could be linked to behavioral stability. A likely explanation for inconsistencies between studies could be the non-unity of executive control functions [39], and their differential demands on cognitive stability and flexibility, which are subserved by neural transmission in different neural structures [40]. Taken together, these studies indicate that A+ and T+ individuals of the DRD2/ANKK1-TaqIA and C957T alleles respectively, demonstrate less cognitive flexibility, but may perform better on tasks taxing cognitive stability, such as cognitive goal maintenance. This hypothesis has not yet been tested.

In the current study we explore the link between striatal dopamine and cognitive goal maintenance in individuals with a genetic predisposition for high compared to low D2 availability. Manipulation of goal maintenance demands was accomplished by using long and short inter-stimulus intervals (ISIs) in the AX-continuous performance task (AX-CPT). We test the hypothesis that a genetic predisposition (C957T T+ and DRD2/ANKK1-TaqIA A+) for reduced D2 receptor density in the striatum would facilitate goal maintenance using the AX-continuous performance task (AX-CPT). We hypothesize that individuals with presumed lower striatal D2 availability would perform selectively better on long ISI BX trials that emphasized cognitive stability/goal maintenance compared to individuals with higher presumed striatal D2 availability, and that this effect would not be present for trials with low demands on goal maintenance, i.e. short ISI BX and AY trials. We believe that our results could have implications for understanding cognitive deficits in patients characterized by altered dopamine functioning, such as schizophrenia, depression, and Parkinson’s disease, and the relationship between dopamine and individual differences in higher cognitive functions in the general population.

## Methods

### Participants

Participants were recruited from the 2010 wave of the Swedish Longitudinal Occupational Survey of Health (SLOSH), which is a longitudinal study of work life, social situation and health among Swedish employees (e.g. [41]). The study is conducted biennially and is approximately representative of the Swedish work force. Participants living in the Stockholm and Gothenburg counties, and their surrounding counties were invited to an in-depth study of cognitive functioning, with testing taking place in Stockholm at the Stress Research Institute, Stockholm University, or in Gothenburg at the Institute for Stress Medicine. A total of 233 people participated in the lab study of cognitive functioning. Exclusion criteria were known or probable brain injury, such as prior head trauma, stroke, or chemical poisoning, as well as other significant illness conditions that can have profound impacts on cognition (e.g., psychotic conditions). Some participants were also excluded because of the lack of genetic data, leaving data from 194 participants for the final genetic analyses (Table 1). The age range was 25–67 years and 75% were women. For more details on sampling procedure and descriptive statistics for the complete sample, see Stenfors et al. [42]. Individuals that agreed to participate were given an appointment for in-person-testing at the lab in their geographical area (Stockholm or Gothenburg). All study participants have given their written informed consent, in accordance with the Declaration of Helsinki. The study was approved by the Ethical Review Board in Stockholm (Dnr 2010/397-31 and 2014/693-32).

**Table 1:** Demographics and off-line cognitive scores.

	0	1	2
Demographics			
N	25	128	41
Age, years (range)	43.2 (24–64)	49.52 (25–67)	49.2 (31–65)
Gender (f/m)	19/6	99/29	27/14
Educational attaintment^a^, (range)	2.2 (1–3)	2.16 (1–3)	2.49 (1–3)
Hypertension^b^	32%	23%	28%
Cognitive scores			
Fluency A (sd)	15.3 (4.9)	14.1 (4.4)	14.1 (4.3)
Fluency B (sd)	7.5 (3.1)	7.2 (2.7)	7.9 (3.3)
Block design (sd)	34.8 (8.5)	33.2 (10.4)	35.3 (9.5)
Stroop—congruent, ms (sd)	610.9 (186.6)	644.6 (216.8)	658.1 (235.2)
Stroop—incongruent, ms (sd)	670.7 (233.1)	757.1 (229.8)	734.4 (192.2)
Stroop—interference, ms (sd)	59.8 (144.9)	112.1 (197.3)	76.3 (162.8)
2-back accuracy, %	0.87 (0.08)	0.87 (0.08)	0.88 (0.08)
2-back reaction time, ms	1134.4 (141)	1115.2 (176)	1143.9 (186)
SRB^c^	24.1 (3.4)	24.1 (3.1)	24.5 (2.9)

^a^ 1 = “up to secondary school/12 years of school”, 2 = university level studies for up to 2 years; to 3 = “more than 2 years of university level studies”;

^b^ Participants who self-reported cardiovascular disease or a hypertensive condition which affects their life in the SLOSH SLOSH (Swedish Longitudinal Occupational Survey of Health) questionnaire or at the lab test occasion (many of which are on anti-hypertensive medication);

^C^Test of vocabulary; Fluency scores reflect number of correctly generated words. Sd = standard deviation.

### Experimental task

Participants performed the AX-CPT is which they were informed that a sequence of letters would be presented and that the task was to press the target “Yes” button (marked out “m” key on a computer keyboard) whenever they saw the letter “X” immediately preceded by the letter “A”. They were also instructed to respond “NO” (marked out “x” key) to all other letters and probes. Each letter was followed by a cross-hair with variable inter-stimulus-interval (ISI), after which the next letter appeared. Participants were asked to respond as quickly and accurately as possible. The letter pairs were presented on a computer display sequentially over 134 trials separated by either a short (1 s) or long (4 s) ISI. Each stimulus remained on the screen for 766 ms. Out of these 134 trials, 90 were AX trials, 20 were AY trials, 20 were BX trials, and 4 were BY trials. Half of the trials were separated by long ISIs and half by short ISIs. Given the small number of BY trials, these were not included in the analyses.

### Data analysis

Analyses of accuracy and reaction times (RT) were conducted in repeated measures analyses of variance (ANOVAs) that focused on comparisons of participant groups: carriers of 0 dopamine alleles (low), carriers of 1 dopamine allele (intermediate), and carriers of 2 dopamine alleles (high). Each ANOVA had group as a between-subjects factor, and delay (short, long) as within-subjects factors. Separate analyses were conducted for target (AX) trials.

### Genotyping

Genotyping was performed on DNA extracted from peripheral blood samples. Samples were labelled anonymously and stored at the Karolinska Biobank before being transferred to the Mutation Analysis Facility at the Karolinska Institute, Huddinge, Sweden, where DNA extraction and genotyping took place. Genotyping was conducted with a single-nucleotide extension reaction, with allele detection by mass spectrometry (Sequenom MassArray system; Sequenom, San Diego, CA USA). PCR and extension primers were designed using the MassArray assay design software. The genotype success rate for SNP rs6277 was 100%. There was no departure from Hardy–Weinberg equilibrium in the sample (p = 0.73). Genotype counts were TT: 54, CT: 99, CC: 41. The genotype success rate for SNP rs1800497 was 100%. The SNP rs1800497 is usually referred to as the TaqIA polymorphism. Participants with the AA and AG genotypes were considered to have A+ allelic status, whereas those with the GG homozygous genotype were considered to have A- allelic status, consistent with previous studies [43] and a dominant model of inheritance. There was no departure from Hardy–Weinberg equilibrium in the sample (*p* = 0.82). Genotype counts were AA: 6, AG: 54, GG: 134. A gene score was calculated based on whether individuals carried 0, 1 or 2 copies of the A (rs1800497) or T (rs6277) allele (rs1800497 GG/rs6277 CC = 0; rs1800497 A+/rs6277 CC *or* rs1800497 GG/rs6277 T+ = 1; rs1800497 A+/rs6277 T+ = 2) associated with lower striatal dopamine availability/receptor density. For control analyses, we also included the COMT gene which has been linked to prefrontal dopamine functioning. The genotypic distribution of COMT Val^158^Met did not deviate significantly from Hardy-Weinberg equilibrium in this sample (P = 0.65). Genotype counts were Met/Met: 63, Val/Met: 100 and Val/Val 45.

## Results

Across genotype groups, and for trials with short ISI, accuracy performance on AY trials was lower compared to AX trials (*t*(230) = 19.7. *P* < 0.001), and performance on BX trials was lower compared to AX trials (*t*(230) = 12.9, *P* < 0.001). There was no significant difference between AY trials and BX trials (*t*(230) = 0.945; *P* = 0.346). For long ISI trials, performance on AY (t(231) = 9.88, P < 0.001) as well as BX trials (t(231) = 10.79, p < 0.001) was significantly lower compared to AX trials. Accuracy for AY trials was significantly higher compared to BX trials (t(231) = 2.25, P = 0.025). The comparison of short vs. long ISI trials showed that accuracy for long ISI AY trials was significantly higher compared to short ISI AY trials (*t*(231) = 3.12, *P* = 0.002), and for AX trials the opposite pattern was found with accuracy for long ISI AX being lower compared with short ISI AX trials (*t*(231) = 2.89, *P* = 0.004). No significant difference between long and short ISI BX trials was found (*t*(231) = 0.13, *P* = 0.898).

**Fig 1:**
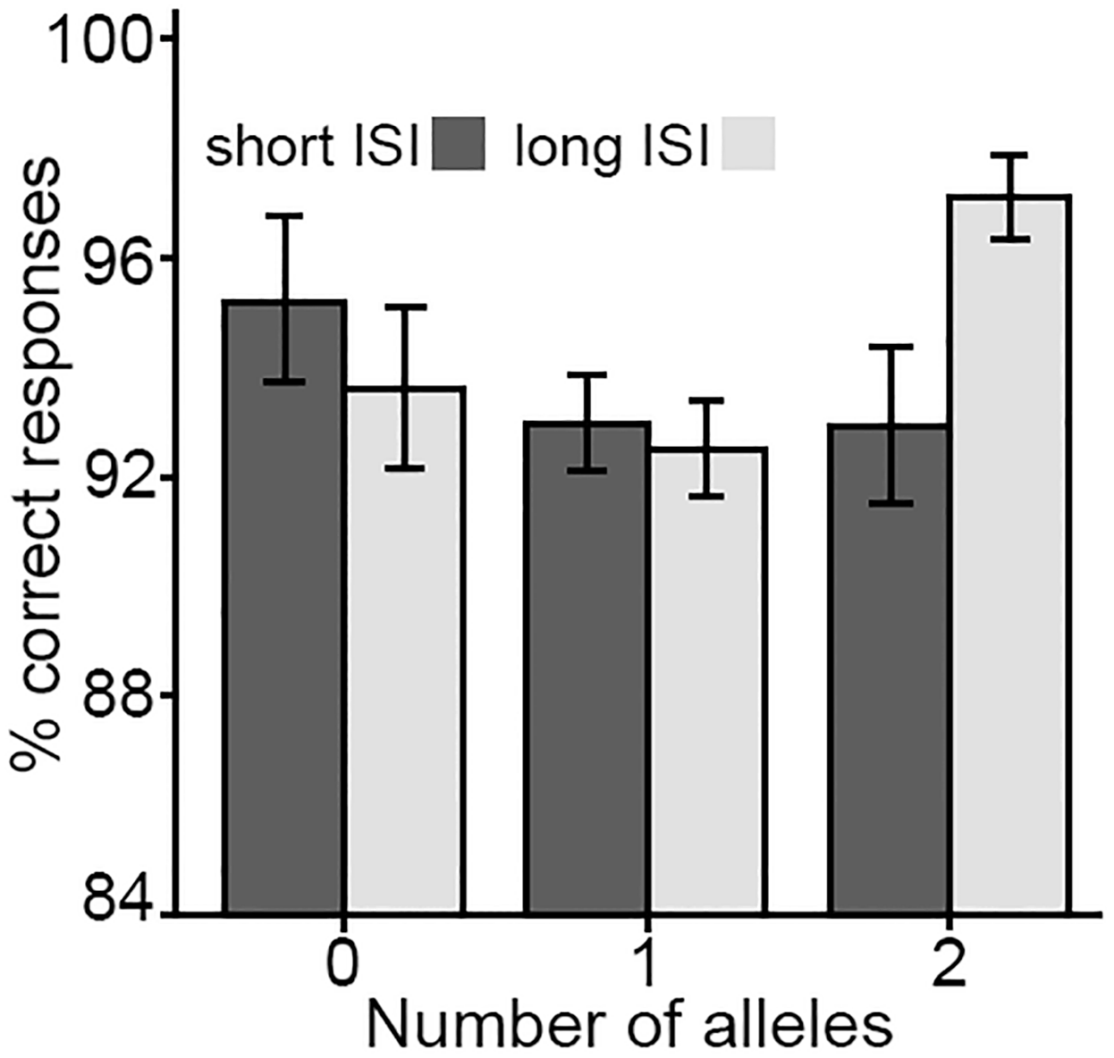
Bar graph showing accuracy (in percent) for BX trials in the AX-CPT. Separate bars are shown for individuals carrying 0, 1, or 2 copies of the A/T allele. Error bars show standard error of the mean.

In order to specifically examine if goal maintenance ability could be predicted from individual genetic variability in DRD2 polymorphisms, we used a repeated measures 3 (genotype group: 0/1/2 copies of the A/T alleles) × 2 (long vs. short ISI) × 3 (trial type: AX, AY vs. BX) ANOVA on accuracy (Fig 1 and S1 data). We hypothesize that individuals with presumed lower striatal D2 availability would perform selectively better on long ISI BX trials that emphasized cognitive stability/goal maintenance compared to individuals with higher presumed striatal D2 availability, and that this effect would not be present for trials with low demands on goal maintenance, i.e. short ISI BX and AY trials. While the main effect of group was non-significant (*F*(1,191) = 2.65; *P* = .073; ƞp2 = .027), the main effects of ISI (*F*(1,191) = 4.28; *P* = .04; ƞp2 = .022) and trial type (*F*(1,191) = 3.66; *P* = .027; ƞp2 = .019) were significant showing that performance for long ISI trials was higher compared to short ISI trials, and that performance for AX trials were higher compared to BX and AY trials. Moreover, the ISI by group interaction was significant showing that the effect of the two DRD2 genes was specific to long ISI trials (*F*(1,191) = 3.89; *P* = .022; ƞp2 = .039). Follow-up analyses showed that the ISI by group interaction was specific to BX trials (*F*(1,191) = 3.49; *P* = .032; ƞp2 = .035), and non-significant for AY trials (*F*(1,191) = 1.24; *P* = .11; ƞp2 = .023).

We also used a repeated measures 3 (genotype group: 0/1/2 copies of the A/T alleles) × 2 (long vs. short ISI) × 3 (trial type: AX, AY, and BX) ANOVA to evaluate reaction time differences between the genotype groups on accurate AX, AY and BX trials to determine whether accuracy differences may be the result of group differences in a speed-accuracy trade-off (Fig 1 and S1 Data). Our analyses indicated a main effect of trial type, where individuals responded faster to AX trials (494 ms) compared to AY (606 ms) and BX trials (597 ms; *F*(1,190) = 141; *P* < .001; ƞp2 = .34). The difference between AY and BX trials across groups and ISI approached significance (*t*(191) = 1.67, P = 0.096). There was also a significant main effect of ISI with RTs being faster on short ISI trials compared to long ISI trials (*F*(1,190) = 6.87; *P* = .009; ƞp2 = .035). We found no interactions between group and trial type (F(1,190) = .37; P = .828; ƞp2 = .004), or ISI (F(1,190) = .78; P = .457; ƞp2 = .008) suggesting that speed-accuracy trade-off differences did not account for the group differences in accuracy described above.

Recent reports have indicated an epistatic interaction between the DRD2/ANKK1 Taq1A polymorphism and the Val158Met SNP of the cathecol-O-methyltransferase (COMT) gene [35,44,45]. The COMT gene is important for dopaminergic activity in the prefrontal cortex [46], and has been associated with working memory processes [47,48]. Therefore, we investigated the independent and interactive contribution of the COMT gene on goal-maintenance. In line with the previous literature, the sample was divided into one group that consisted of individuals carrying one or two copies of the MET allele (high frontal dopamine) and one group that included VAL/VAL carriers (low frontal dopamine). First, we did not find any significant main effects or interactions of the COMT gene on AX-CPT performance (all *F*s < 1). Second, we performed control analyses where we included COMT as a covariate in the analyses of the DRD2 genes. Most importantly, we were able to replicate the significant DRD2 gene by ISI interaction on BX trials reported above, suggesting that this effect was not mediated by individual differences in prefrontal dopamine functioning associated with the COMT gene.

Moreover, all significant main findings were replicated when age and sex were used as covariates in the analyses.

## Discussion

Using the AX-CPT we demonstrate that carriers of two polymorphisms that have been linked to reduced striatal D2 receptor density show increased performance on context-dependent (BX) trials, and that the effect of these polymorphisms was only significant for long ISI trials where the demand for goal maintenance is high. Group differences were not related to participants’ age or sex, and the effect was not modulated by the COMT genotype. This study provides first evidence of enhanced goal-maintenance in carriers of dopamine polymorphisms associated with reduced striatal dopamine availability.

The present results are in line with the notion that the balance between tonic and phasic dopamine neurotransmission is critical for optimal neurocognitive functioning. Our results also support the notion that tonic and phasic modulation of dopamine have specific effects on different aspects of cognition; tonic stimulation of dopamine D1 receptors help maintain and stabilize relevant information, while phasic activation of dopamine D2 receptors is associated with cognitive flexibility including manipulation and updating of working memory representations [7,40,49]. Our results suggests that DRD2 polymorphisms may alter the balance between tonic and phasic neurotransmission by showing that alleles associated with reduced striatal dopamine availability are related to superior cognitive stability as indicated by greater goal maintenance, which depend on tonic neurotransmission. Critically, the effect of the DRD2 genes was only found for long ISI, as opposed to short ISI BX trials, further underscoring that the effect was specific to trials for which the demands on goal maintenance processes were high.

The current results nicely aligns with previous findings showing that carriers of the ANKK1/DRD2-Taq1a A1 allele, with presumably reduced D2 receptor density, perform at a lower level on tasks requiring updating and cognitive flexibility [31,33], indicating increased cognitive stability in these participants. Our results extend these previous findings by showing that participants with lower D2 receptor density are better at goal maintenance, a task with high demands on cognitive stability, and further suggests that high DRD2 density may be disadvantageous for certain cognitive operations. Similarly, the ANKK1-Taq1A polymorphism, in epistatic interaction with the COMT gene, has been linked to updating of goal representations suggesting that low D2 availability may be associated with WM maintenance and higher cognitive stability [35]. The present findings thus indicate that individuals with lower D2 receptor density may perform better in tasks that require maintenance while performing worse in tasks that require updating and cognitive flexibility. Current results also indirectly supports the findings from a previous study [34] showing that older individuals with higher D2 receptor density had higher updating performance and concomitant BOLD signal change in the caudate nucleus compared to individuals with lower D2 receptor density suggesting that less available dopamine may be linked to reduced flexibility and higher stability.

Moreover, it has been demonstrated that homozygous carriers of the C957T T-polymorphism, which has been linked to reduced striatal D2 availability, performed at a lower level on a 2-back WM task that requires updating, and that this group had larger beneficial effect of dopamine supplementation compared to CC homozygotes [50]. This suggests that higher D2 DA levels in striatum can be related to over-flexible behavior and reduced maintenance of contexts and goal states. It has also been shown, using a rapid serial visual presentation task, that the attentional blink is reduced in DRD2 C957T T/T homozygotes, indicating superior efficiency in preventing targets from being overwritten by distractors [51]. In line with the current findings showing that C957T T-carriers show greater behavioral stability as indicated by increased goal maintenance compared with non-carriers, the authors argue that C957T T/T homozygotes may be more efficient in suppressing irrelevant information, such as the nontargets in an AB task.

Our findings also corroborate findings of impaired goal maintenance performance in patients with dopaminergic dysregulation, such as patients with schizophrenia. For example, presynaptic dopaminergic hyperactivity is a common finding in neurochemical imaging studies of schizophrenia. This observation is based on the repeatedly demonstrated increase of striatal dopamine (DA) synthesis capacity in studies using radiolabeled L-DOPA, the precursor of DA. The evidence of striatal dopamine hyperactivity in schizophrenia is further strengthened by findings of increases in baseline DA synaptic concentration and release using D2 receptors imaging [52]. Importantly, numerous studies have provided evidence that individuals with schizophrenia have difficulties with goal maintenance in WM. Such deficits are present in both medicated and unmedicated individuals, and at both acute and chronic stages of the illness [53–57], and thus suggest a link between high striatal levels of dopamine and deficits in goal maintenance.

The lack of any effects of COMT on AX-CPT performance was somewhat surprising given findings of interactions between the DRD2/ANKK1-Taq1A polymorphism and the COMT gene [35,44,45], and its association with working memory processes [47,48]. However, the negative finding for an effect of the COMT Val158Met allele on AX-CPT performance is in line with several previous studies, including a meta-analysis by Barnett et al. [58], which concluded that COMT Val158Met has little if any effect on cognitive function. It should also be noted that the number of participants with a genetic predisposition for reduced striatal D2 receptor availability (C957T T+ and DRD2/ANKK1-TaqIA A+), together with the MET allele (increased frontal dopamine availability) was rather small, thus reducing power to detect any potential interactive effects of these alleles.

It is important to emphasize that while the observed effects of these two DRD2 polymorphisms were robust and selective to goal maintenance, these SNPs accounted for only around 4% of the total variance. It should be noted, however, that executive function is a complex trait, and it is therefore expected to be supported by many genes, each of which contribute with a relatively small effect. Despite its modest individual contribution, small effects of multiple factors can still exert a meaningful influence on complex cognitive traits such as executive function and working memory. Future studies that includes larger samples could contribute valuable information by investigating the contribution of gene polymorphisms on complex traits using polygenic risk scores based on case-control data from large-scale genome-wide association studies.

In the current study we provide first evidence of a link between genetic inter-individual variation in D2 receptor expression and goal-maintenance. We show that carriers of two genes associated with lower striatal dopamine receptor density perform at a higher level compared to non-carriers, and that this difference is specific to trials that require maintenance of active goal representations. These results add further knowledge to a more general framework concerning the role of D2 receptor functioning in cognitive stability and flexibility.

## Supporting information

S1 Data Behavioral data. A file containing behavioral data from the AX-CPT task along with demographic information for each of the two genetic groups.
(XLSX)
